# Fundamentals and Methods for T- and B-Cell Epitope Prediction

**DOI:** 10.1155/2017/2680160

**Published:** 2017-12-28

**Authors:** Jose L. Sanchez-Trincado, Marta Gomez-Perosanz, Pedro A. Reche

**Affiliations:** Laboratory of Immunomedicine, Faculty of Medicine, Complutense University of Madrid, Ave Complutense S/N, 28040 Madrid, Spain

## Abstract

Adaptive immunity is mediated by T- and B-cells, which are immune cells capable of developing pathogen-specific memory that confers immunological protection. Memory and effector functions of B- and T-cells are predicated on the recognition through specialized receptors of specific targets (antigens) in pathogens. More specifically, B- and T-cells recognize portions within their cognate antigens known as epitopes. There is great interest in identifying epitopes in antigens for a number of practical reasons, including understanding disease etiology, immune monitoring, developing diagnosis assays, and designing epitope-based vaccines. Epitope identification is costly and time-consuming as it requires experimental screening of large arrays of potential epitope candidates. Fortunately, researchers have developed in silico prediction methods that dramatically reduce the burden associated with epitope mapping by decreasing the list of potential epitope candidates for experimental testing. Here, we analyze aspects of antigen recognition by T- and B-cells that are relevant for epitope prediction. Subsequently, we provide a systematic and inclusive review of the most relevant B- and T-cell epitope prediction methods and tools, paying particular attention to their foundations.

## 1. Introduction

The immune system is typically divided into two categories, innate and adaptive. Innate immunity involves nonspecific defense mechanisms that act immediately or within hours after a microbe appearance in the body. All multicellular beings exhibit some kind of innate immunity. In contrast, adaptive immunity is only present in vertebrates and it is highly specific. In fact, the adaptive immune system is able to recognize and destroy invading pathogens individually. Moreover, the adaptive immune system remembers the pathogens that fights, acquiring a pathogen-specific long-lasting protective memory that enables stronger attacks each time the pathogen is reencountered [1]. Nonetheless, innate and adaptive immune mechanisms work together and adaptive immunity elicitation is contingent on prior activation of innate immune responses [1].

Adaptive immunity is articulated by lymphocytes, more specifically by B- and T-cells, which are responsible for the humoral and cell-mediated immunity. B- and T-cells do not recognize pathogens as a whole, but molecular components known as antigens. These antigens are recognized by specific receptors present in the cell surface of B- and T-cells. Antigen recognition by these receptors is required to activate B- and T-cells but not enough, as second activation signals stemming from the activation of the innate immune system are also needed. The specificity of the recognition is determined by genetic recombination events that occur during lymphocyte development, which lead to generating millions of different variants of lymphocytes in terms of the antigen-recognizing receptors [1]. Antigen recognition by B- and T-cells differ greatly.

**Figure 1:**
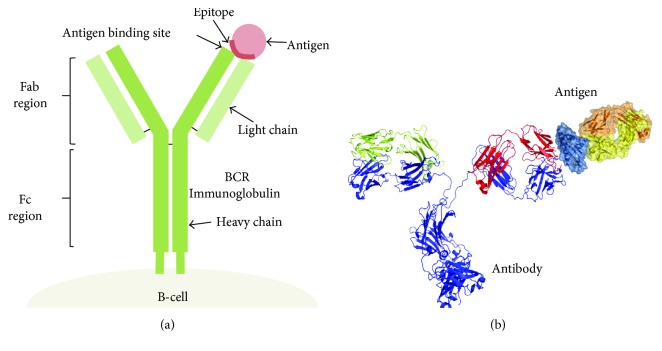
B-cell epitope recognition. B-cell epitopes are solvent-exposed portions of the antigen that bind to secreted and cell-bound immunoglobulins. (a) B-cell receptors encompass cell-bound immunoglobulins, consisting of two heavy chains and two light chains. The different chains and regions are annotated. (b) Molecular representation of the interaction between an antibody and the antigen. Antibodies are secreted immunoglobulins of known specificity.

B-cells recognize solvent-exposed antigens through antigen receptors, named as B-cell receptors (BCR), consisting of membrane-bound immunoglobulins, as shown in Figure 1. Upon activation, B-cells differentiate and secrete soluble forms of the immunoglobulins, also known as antibodies, which mediate humoral adaptive immunity. Antibodies released by B-cells can have different functions that are triggered upon binding their cognate antigens. These functions include neutralizing toxins and pathogens and labeling them for destruction [1].

A B-cell epitope is the antigen portion binding to the immunoglobulin or antibody. These epitopes recognized by B-cells may constitute any exposed solvent region in the antigen and can be of different chemical nature. However, most antigens are proteins and those are the subjects for epitope prediction methods.

**Figure 2:**
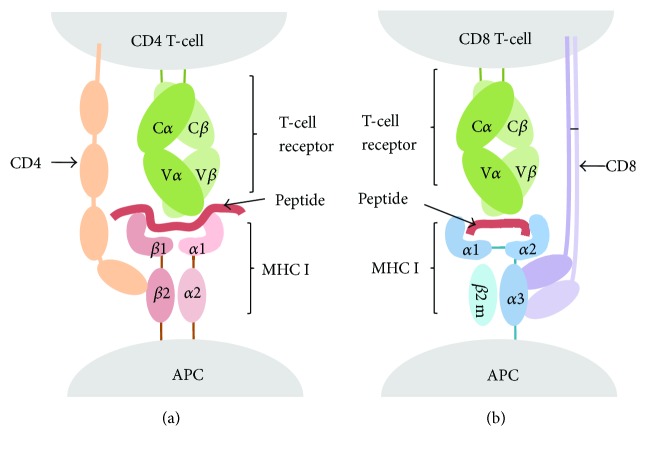
T-cell epitope recognition. T-cell epitopes are peptides derived from antigens and recognized by the T-cell receptor (TCR) when bound to MHC molecules displayed on the cell surface of APCs. (a) CD4 T-cells express the CD4 coreceptor, which binds to MHC II, and recognize peptides presented by MHC II molecules. (b) CD8 T-cells express the CD8 coreceptor, which binds to MHC I, and recognize peptides presented by MHC I molecules.

On the other hand, T-cells present on their surface a specific receptor known as T-cell receptor (TCR) that enables the recognition of antigens when they are displayed on the surface of antigen-presenting cells (APCs) bound to major histocompatibility complex (MHC) molecules. T-cell epitopes are presented by class I (MHC I) and II (MHC II) MHC molecules that are recognized by two distinct subsets of T-cells, CD8 and CD4 T-cells, respectively (Figure 2). Subsequently, there are CD8 and CD4 T-cell epitopes. CD8 T-cells become cytotoxic T lymphocytes (CTL) following T CD8 epitope recognition. Meanwhile, primed CD4 T-cells become helper (Th) or regulatory (Treg) T-cells [1]. Th cells amplify the immune response, and there are three main subclasses: Th1 (cell-mediated immunity against intracellular pathogens), Th2 (antibody-mediated immunity), and Th17 (inflammatory response and defense against extracellular bacteria) [2].

Identifying epitopes in antigens is of great interest for a number of practical reasons, including understanding disease etiology, immune monitoring, developing diagnosis assays, and designing epitope-based vaccines. B-cell epitopes can be identified by different methods including solving the 3D structure of antigen-antibody complexes, peptide library screening of antibody binding or performing functional assays in which the antigen is mutated and the interaction antibody-antigen is evaluated [3, 4]. On the other hand, experimental determination of T-cell epitopes is carried out using MHC multimers and lymphoproliferation or ELISPOT assays, among others [5, 6]. Traditional epitope identification has depended entirely upon experimental techniques, being costly and time-consuming. Thereby, scientists have developed and implemented epitope prediction methods that facilitate epitope identification and decrease the experimental load associated with it. Here, we will first analyze aspects of antigen recognition by T- and B-cells that are relevant for a better understanding of the topic of epitope prediction. Subsequently, we will provide a systematic and inclusive review of the most important prediction methods and tools, paying particular attention to their foundations and potentials. We will also discuss epitope prediction limitations and ways to overcome them. We will start with T-cell epitopes.

## 2. T-Cell Epitope Prediction

T-cell epitope prediction aims to identify the shortest peptides within an antigen that are able to stimulate either CD4 or CD8 T-cells [7]. This capacity to stimulate T-cells is called immunogenicity, and it is confirmed in assays requiring synthetic peptides derived from antigens [5, 6]. There are many distinct peptides within antigens and T-cell prediction methods aim to identify those that are immunogenic. T-cell epitope immunogenicity is contingent on three basic steps: (i) antigen processing, (ii) peptide binding to MHC molecules, and (iii) recognition by a cognate TCR. Of these three events, MHC-peptide binding is the most selective one at determining T-cell epitopes [8, 9]. Therefore, prediction of peptide-MHC binding is the main basis to anticipate T-cell epitopes and we will review it next.

### 2.1. Prediction of Peptide-MHC Binding

**Figure 3:**
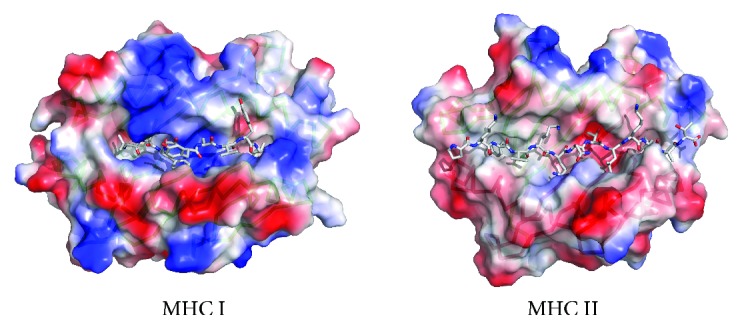
MHC molecule binding groove. The figure depicts the molecular surface as seen by the TCR of representative MHC I and II molecules. Note how the binding groove of the MHC I molecule is closed but that of MHC II is open. As a result, MHC I molecules bind short peptides (8–11 amino acids), while MHC II molecules bind longer peptides (9–22 amino acids). The figure was prepared from PDB files 1QRN (MHC I) and 1FYT (MHC II) using PyMol.

MHC I and MHC II molecules have similar 3D-structures with bound peptides sitting in a groove delineated by two *α*-helices overlying a floor comprised of eight antiparallel *β*-strands. However, there are also key differences between MHC I and II binding grooves that we must highlight for they condition peptide-binding predictions (Figure 3). The peptide-binding cleft of MHC I molecules is closed as it is made by a single *α* chain. As a result, MHC I molecules can only bind short peptides ranging from 9 to 11 amino acids, whose N- and C-terminal ends remain pinned to conserved residues of the MHC I molecule through a network of hydrogen bonds [10, 11]. The MHC I peptide-binding groove also contains deep binding pockets with tight physicochemical preferences that facilitate binding predictions. There is a complication however. Peptides that have different sizes and bind to the same MHC I molecule often use alternative binding pockets [12]. Therefore, methods predicting peptide-MHC I binding require a fixed peptide length. However, since most MHC I peptide ligands have 9 residues, it is generally preferable to predict peptides with that size. In contrast, the peptide-binding groove of MHC II molecules is open, allowing the N- and C-terminal ends of a peptide to extend beyond the binding groove [10, 11]. As a result, MHC II-bound peptides vary widely in length (9–22 residues), although only a core of nine residues (peptide-binding core) sits into the MHC II binding groove. Therefore, peptide-MHC II binding prediction methods often target to identify these peptide-binding cores. MHC II molecule binding pockets are also shallower and less demanding than those of MHC I molecules. As a consequence, peptide-binding prediction to MHC II molecules is less accurate than that of MHC I molecules.

Given the relevance of the problem, there are numerous methods to predict peptide-MHC binding. The most relevant with free online use are collected on Table 1. They can be divided in two main categories: data-driven and structure-based methods. Structure-based approaches generally rely on modeling the peptide-MHC structure followed by evaluation of the interaction through methods such as molecular dynamic simulations [8, 13]. Structure-based methods have the great advantage of not needing experimental data. However, they are seldom used as they are computationally intensive and exhibit lower predictive performance than data-driven methods [14].

Data-driven methods for peptide-MHC binding prediction are based on peptide sequences that are known to bind to MHC molecules. These peptide sequences are generally available in specialized epitope databases such as IEDB [15], EPIMHC [16], Antijen [17, 18]. Both MHC I and II binding peptides contain frequently occurring amino acids at particular peptide positions, known as anchor residues. Thereby, prediction of peptide-MHC binding was first approached using sequence motif (SM) reflecting amino acid preferences of MHC molecules at anchor positions [19]. However, it was soon shown that nonanchor residues also contribute to the capacity of a peptide to bind to a given MHC molecule [20, 21]. Subsequently, researchers developed motif matrices (MM), which could evaluate the contribution of each and all peptide positions to the binding with the MHC molecule [22–25]. The most sophisticated form of motif matrices consists of profiles [24–26] that are similar to those used for detecting sequence homology [27]. We would like to remark that motif matrices are often mistaken with quantitative affinity matrices (QAMs) since both produce peptide scores. However, MMs are derived without taking in consideration values of binding affinities and, therefore, resulting peptide scores are not suited to address binding affinity. In contrast, QAMs are trained on peptides and corresponding binding affinities, and aim to predict binding affinity. The first method based on QAMs was developed by Parker et al. [28] (Table 1). Subsequently, various approaches were developed to obtain QAMs from peptide affinity data and predict peptide binding to MHC I and II molecules [29–32].

QAMs and motif matrices assume an independent contribution of peptide side chains to the binding. This assumption is well supported by experimental data but there is also evidence that neighboring peptide residues interfere with others [33]. To account for those interferences, researchers introduced quantitative structure-activity relationship (QSAR) additive models wherein the binding affinity of peptides to MHC is computed as the sum of amino acid contributions at each position plus the contribution of adjacent side chain interactions [34]. However, machine learning (ML) is the most popular and robust approach introduced to deal with the nonlinearity of peptide-MHC binding data [8]. Researchers have used ML for two distinct problems: the discrimination of MHC binders from nonbinders and the prediction of binding affinity of peptides to MHC molecules.

For developing discrimination models, ML algorithms are trained on data sets consisting of peptides that either bind or do not bind to MHC molecules. Relevant examples of ML-based discrimination models are those based on artificial neural networks (ANNs) [35, 36], support vector machines (SVMs) [37–39], decision trees (DTs) [40, 41], and Hidden Markov models (HMMs), which can also cope with nonlinear data and have been used to discriminate peptides binding to MHC molecules. However, unlike other ML algorithms, they have to be trained only on positive data. Three types of HMMs have been used to predict MHC-peptide binding: fully connected HMMs [42], structure-optimized HMMs [43], and profile HMMs [43, 44]. Of these, only fully connected HMMs (fcHMMs) and structure-optimized HMMs (soHMMs) can recognize different patterns in the peptide binders. In fact, profile HMMs that are derived from sets of ungapped alignments (the case for peptides binding to MHC) are nearly identical to profile matrices [45] (Table 1).

With regard to predicting binding affinity, ML algorithms are trained on datasets consisting of peptides with known affinity to MHC molecules. Both SVMs and ANNs have been used for such purpose. SVMs were first applied to predict peptide-binding affinity to MHC I molecules [46] and later to MHC II molecules [47] (Table 1). Likewise, ANNs were also applied first to the prediction of peptide binding to MHC I [48, 49] and later to MHC II molecules [50] (Table 1). Benchmarking of peptide-MHC binding prediction methods appears to indicate that those based on ANNs are superior to those based on QAMs and MMs. However, the differences between the distinct methods are marginal and vary for different MHC molecules [51]. Moreover, it has been shown that the performance of peptide-MHC predictions is improved by combining several methods and providing consensus predictions [52].

A major complication for predicting T-cell epitopes through peptide-MHC binding models is MHC polymorphism. In humans, MHC molecules are known as human leukocyte antigens (HLAs), and there are hundreds of allelic variants of class I (HLA I) and class II (HLA II) molecules. These HLA allelic variants bind distinct sets of peptides [53] and require specific models for predicting peptide-MHC binding. However, peptide-binding data is only available for a minority of HLA molecules. To overcome this limitation, some researchers have developed pan-MHC-specific methods by training ANNs on input data combining MHC residues that contact the peptide with peptide-binding affinity that are capable of predicting peptide-binding affinities to uncharacterized HLA alleles [54, 55].

HLA polymorphism also hampers the development of worldwide covering T-cell epitope-based vaccines as HLA variants are expressed at vastly variable frequencies in different ethnic groups [56]. Interestingly, different HLA molecules can also bind similar sets of peptides [57, 58] and researchers have devised methods to cluster them in groups, known as HLA supertypes, consisting of HLA alleles with similar peptide-binding specificities [59–61]. The HLA-A2, HLA-A3, and HLA-B7 are relevant examples of supertypes; 88% of the population expresses at least an allele included in these supertypes [25, 57, 58]. Identification of promiscuous peptide-binding to HLA supertypes enables the development of T-cell epitope vaccines with high-population coverage using a limited number of peptides. Currently, several web-based methods allow the prediction of promiscuous peptide-binding to HLA supertypes for epitope vaccine design including MULTIPRED [62] and PEPVAC [63] (Table 1). A method to identify promiscuous peptide-binding beyond HLA supertypes was developed and implemented by Molero-Abraham et al. [64] with the name of EPISOPT. EPISOPT predicts HLA I presentation profiles of individual peptides regardless of supertypes and identifies epitope combinations providing a wider population protection coverage.

Prediction of peptide binding to MHC II molecules readily discriminate CD4 T-cell epitopes, but cannot tell their ability to activate the response of specific CD4 T-cell subsets (e.g., Th1, Th2, and Treg). However, there is evidence that some CD4 T-cell epitopes appear to stimulate specific subsets of Th cells [65, 66]. Distinguishing the ability of MHC II-restricted epitopes to elicit distinct responses is clearly relevant for epitope vaccine development and has prompted researchers' attention. A relevant example is the work by Dhanda et al. [67] who generated classifiers capable of predicting potential peptide inducers of interleukin 4 (IL-4) secretion, typical of Th2 cells, by training SVM models on experimentally validated IL4 inducing and noninducing MHC class II binders (Table 1).

### 2.2. Prediction of Antigen Processing and Integration with Peptide-MHC Binding Prediction

**Figure 4:**
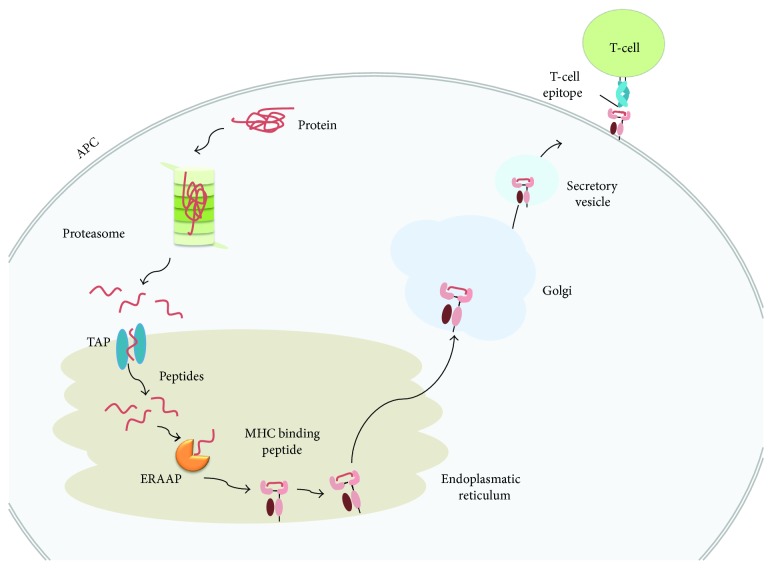
Class I antigen processing. The figure depicts the major steps involved in antigen presentation by MHC I molecules. Proteins are degraded by the proteasome and peptide fragments transported to the endoplasmic reticulum (ER) by TAP where they are loaded onto nascent MHC I molecules. TAP transports peptides ranging from 8 to 16 amino acids. Long peptides cannot bind MHC I molecules but often become suitable for binding after N-terminal trimming by ERAAP.

Antigen processing shapes the peptide repertoire available for MHC binding and is a limiting step determining T-cell epitope immunogenicity [68]. Subsequently, computational modeling of the antigen processing pathway provides a mean to enhance T-cell epitope predictions. Antigen presentation by MHC I and II molecules proceed by two different pathways. MHC II molecules present peptide antigens derived from endocyted antigens that are degraded and loaded onto the MHC II molecule in endosomal compartments [69]. Class II antigen degradation is poorly understood, and there is lack of good prediction algorithms yet [70]. In contrast, MHC I molecules present peptides derived mainly from antigens degraded in the cytosol. The resulting peptide antigens are then transported to the endoplasmic reticulum by TAP where they are loaded onto nascent MHC I molecules [69] (Figure 4). Prior to loading, peptides often undergo trimming by ERAAP N-terminal amino peptidases [71].

Proteasomal cleavage and peptide-binding to TAP have been studied in detail and there are computational methods that predict both processes. Proteasomal cleavage prediction models have been derived from peptide fragments generated *in vitro* by human constitutive proteasomes [72, 73] and from sets of MHC I-restricted ligands mapped onto their source proteins [74–76]. On the other hand, TAP binding prediction methods have been developed by training different algorithms on peptides of known affinity to TAP [77–80]. Combination of proteasomal cleavage and peptide-binding to TAP with peptide-MHC binding predictions increases T-cell epitope predictive rate in comparison to just peptide-binding to MHC I [37, 77, 81–83]. Subsequently, researchers have developed resources to predict CD8 T-cell epitopes through multistep approaches integrating proteasomal cleavage, TAP transport, and peptide-binding to MHC molecules [26, 37, 82–85] (Table 1).

## 3. Prediction of B-Cell Epitopes

**Figure 5:**
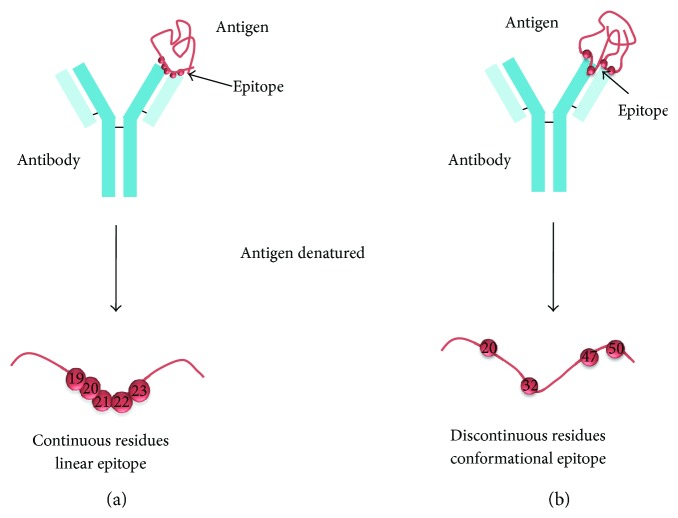
Linear and conformational B-cell epitopes. Linear B-cell epitopes (a) are composed of sequential/continuous residues, while conformational B-cell epitopes (b) contain scattered/discontinuous residues along the sequence.

B-cell epitope prediction aims to facilitate B-cell epitope identification with the practical purpose of replacing the antigen for antibody production or for carrying structure-function studies. Any solvent-exposed region in the antigen can be subject of recognition by antibodies. Nonetheless, B-cell epitopes can be divided in two main groups: linear and conformational (Figure 5). Linear B-cell epitopes consist of sequential residues, peptides, whereas conformational B-cell epitopes consist of patches of solvent-exposed atoms from residues that are not necessarily sequential (Figure 5). Therefore, linear and conformational B-cell epitopes are also known as continuous and discontinuous B-cell epitopes, respectively. Antibodies recognizing linear B-cell epitopes can recognize denatured antigens, while denaturing the antigen results in loss of recognition for conformational B-cell epitopes. Most B-cell epitopes (approximately a 90%) are conformational and, in fact, only a minority of native antigens contains linear B-cell epitopes [3]. We will review both, prediction of linear and conformational B-cell epitopes.

### 3.1. Prediction of Linear B-Cell Epitopes

Linear B-cell epitopes consist of peptides which can readily be used to replace antigens for immunizations and antibody production. Therefore, despite being a minority, prediction of linear B-cell epitopes have received major attention. Linear B-cell epitopes are predicted from the primary sequence of antigens using sequence-based methods. Early computational methods for the prediction of B-cell epitopes were based on simple amino acid propensity scales depicting physicochemical features of B-cellepitopes. For example, Hopp and Wood applied residue hydrophilicity calculations for B-cell epitope prediction [96, 97] on the assumption that hydrophilic regions are predominantly located on the protein surface and are potentially antigenic. We know now, however, that protein surfaces contain roughly the same number of hydrophilic and hydrophobic residues [98]. Other amino acid propensity scales introduced for B-cell epitope prediction are based on flexibility [99], surface accessibility [100], and *β*-turn propensity [101]. Current available bioinformatics tools to predict linear B-cell epitopes using propensity scales include PREDITOP [102] and PEOPLE [103] (Table 2). PREDITOP [102] uses a multiparametric algorithm based on hydrophilicity, accessibility, flexibility, and secondary structure properties of the amino acids. PEOPLE [103] uses the same parameters and in addition includes the assessment of *β*-turns. A related method to predict B-cell epitopes was introduced by Kolaskar and Tongaonkar [104], consisting on a simple antigenicity scale derived from physicochemical properties and frequencies of amino acids in experimentally determined B-cell epitopes. This index is perhaps the most popular antigenic scale for B-cell epitope prediction, and it is actually implemented by GCG [105] and EMBOSS [106] packages. Comparative evaluations of propensity scales carried out in a dataset of 85 linear B-cell epitopes showed that most propensity scales predicted between 50 and 70% of B-cell epitopes, with the *β*-turn scale reaching the best values [101, 107]. It has also been shown that combining the different scales does not appear to improve predictions [102, 108]. Moreover, Blythe and Flower [109] demonstrated that single-scale amino acid propensity scales are not reliable to predict epitope location.

The poor performance of amino acid scales for the prediction of linear B-cell epitopes prompted the introduction of machine learning- (ML-) based methods (Table 2). These methods are developed by training ML algorithms to distinguish experimental B-cell epitopes from non-B-cell epitopes. Prior to training, B-cell epitopes are translated into feature vectors capturing selected properties, such as those given by different propensity scales. Relevant examples of B-cell epitope prediction methods based on ML include BepiPred [110], ABCpred [111], LBtope [112], BCPREDS [113], and SVMtrip [114]. Datasets, training features, and algorithms used for developing these methods differ. BepiPred is based on random forests trained on B-cell epitopes obtained from 3D-structures of antigen-antibody complexes [110]. Both BCPREDS [113] and SVMtrip [114] are based on support vector machines (SVM) but while BCPREDS was trained using various string kernels that eliminate the need for representing the sequence into length-fixed feature vectors, SMVtrip was trained on length-fixed tripeptide composition vectors. ABCpred and LBtope methods consist on artificial neural networks (ANNs) trained on similar positive data, B-cell epitopes, but differ on negative data, non-B-cell epitopes. Negative data used for training ABCpred consisted on random peptides while negative data used for LBtope was based on experimentally validated non-B-cell epitopes form IEDB [15]. In general, B-cell epitope prediction methods employing ML-algorithm are reported to outperform those based on amino acid propensity scales. Nevertheless, some authors have reported that ML algorithms show little improvement over single-scale-based methods [115].

Antibodies elicited in the course of an immune response are generally of a given isotype that determines their biological function. A recent advance in B-cell epitope prediction is the development of a method by Gupta et al. [116] that allows the identification of B-cell epitopes capable of inducing specific class of antibodies. This method is based on SMVs trained on a dataset that includes linear B-cell epitopes known to induce IgG, IgE, and IgA antibodies.

### 3.2. Prediction of Conformational B-Cell Epitopes

Most B-cell epitopes are conformational and yet, prediction of conformational B-cell epitopes has lagged behind that of linear B-cell epitopes. There are two main practical reasons for that. First of all, prediction of conformational B-cell epitopes generally requires the knowledge of protein three-dimensional (3D) structure and this information is only available for a fraction of proteins [117]. Secondly, isolating conformational B-cell epitopes from their protein context for selective antibody production is a difficult task that requires suitable scaffolds for epitope grafting. Thereby, prediction of conformational B-cell prediction is currently of little relevance for epitope vaccine design and antibody-based technologies. Nonetheless, prediction of conformational B-cell epitopes is interesting for carrying structure-function studies involving antibody-antigen interactions.

There are several available methods to predict conformational B-cell epitopes (Table 2). The first to be introduced was CEP [118], which relied almost entirely on predicting patches of solvent-exposed residues. It was followed by DiscoTope [119], which, in addition to solvent accessibility, considered amino acid statistics and spatial information to predict conformational B-cell epitopes. An independent evaluation of these two methods using a benchmark dataset of 59 conformational epitopes revealed that they did not exceed a 40% of precision and a 46% of recall [120]. Subsequently, more methods were developed, like ElliPro [121] that aims to identify protruding regions in antigen surfaces and PEPITO [122] and SEPPA [123] that combine single physicochemical properties of amino acids and geometrical structure properties. The reported area under the curve (AUC) of these methods is around 0.7, which is indicative of a poor discrimination capacity yet better than random. Though, in an independent evaluation, SEPPA reached an AUC of 0.62 while all the mentioned methods had an AUC around 0.5 [124]. ML has also been applied to predict conformational B-cell epitopes in 3D-structures. Relevant examples include EPITOPIA [125] and EPSVR [126] which are based on naïve Bayes classifiers and support vector regressions, respectively, trained on feature vectors combining different scores. The reported AUC of these two methods is around 0.6.

The above methods for conformational B-cell epitope prediction identify generic antigenic regions regardless of antibodies, which are ignored [127]. However, there are also methods for antibody-specific epitope prediction. This approach was pioneered by Soga et al. [128] who defined an antibody-specific epitope propensity (ASEP) index after analyzing the interfaces of antigen-antibody 3D-structures. Using this index, they developed a novel method for predicting epitope residues in individual antibodies that worked by narrowing down candidate epitope residues predicted by conventional methods. More recently, Krawczyk et al. [129] developed EpiPred, a method that uses a docking-like approach to match up antibody and antigen structures, thus identifying epitope regions on the antigen. A similar approach is used by PEASE [130], adding that this method utilizes the sequence of the antibody and the 3D-structure of the antigen. Briefly, for each pair of antibody sequence and antigen structure, PEASE uses a machine learning model trained on properties from 120 antibody-antigen complexes to identify pair combination of residues from complementarity-determining regions (CDRs) of the antibody and the antigen that are likely to interact.

Another approach to identify conformational B-cell epitopes in a protein with a known 3D-structure is through mimotope-based methods. Mimotopes are peptides selected from randomized peptide libraries for their ability to bind to an antibody raised against a native antigen. Mimotope-based methods require to input antibody affinity-selected peptides and the 3D-structure of the selected antigen. Examples of bioinformatics tools for conformational B-cell epitope prediction using mimotopes include MIMOX [131], PEPITOPE [132], EPISEARCH [133], MIMOPRO [134], and PEPMAPPER [135] (Table 2).

As remarked before, methods for conformational B-cell epitope prediction generally require the 3D-structure of the antigen. Exceptionally, however, Ansari and Raghava [136] developed a method (CBTOPE) for the identification of conformational B-cell epitope from the primary sequence of the antigen. CBTOPE is based on SVM and trained on physicochemical and sequence-derived features of conformational B-cell epitopes. CBTOPE reported accuracy was 86.6% in crossvalidation experiments.

## 4. Concluding Remarks

Currently, T-cell epitope prediction is more advanced and reliable than that of B-cell prediction. However, while it is possible to confirm experimentally the predicted binding to MHC molecules of most peptides predicted, only ~10% of those are shown to be immunogenic (able to elicit a T-cell response) [68]. Such a low T-cell epitope discovery rate is due to the fact that we do not have adequate models for predicting antigen processing yet [68]. The economic toll of low T-cell epitope discovery rate can be overcome, at least in part, by prioritizing protein antigens for epitope prediction [137–139]. For T-cell epitope vaccine development, researchers can also resort to experimentally known T-cell epitopes, available in epitope databases, selecting through immunoinformatics those that provide maximum population protection coverage [64, 140, 141]. In any case, T-cell epitope prediction remains an integral part of T-cell epitope mapping approaches. In contrast, B-cell epitope prediction utility is currently much more limited. There are several reasons to that. First of all, prediction of B-cell epitopes is still unreliable for both linear and conformational B-cell epitopes. Secondly, linear B-cell epitopes do usually elicit antibodies that do not crossreact with native antigens. Third, the great majority of B-cell epitopes are conformational and yet predicting conformational epitopes have few applications, as they cannot be isolated from their protein context. Under this scenario, the key is not only to improve current methods for B-cell epitope prediction but also to develop novel approaches and platforms for epitope grafting onto suitable scaffolds capable of replacing the native antigen.

To conclude, we wish to make two final remarks that are relevant for epitope vaccine design. First of all, it is that epitope prediction methods can provide potential epitopes from any given protein query but not all the antigens are equally relevant for vaccine development. Therefore, researchers have also developed tools to identify vaccine candidate antigens [142, 143], those likely to induce protective immunity, which can then be targeted for epitope prediction and epitope vaccine design. Second, it should be borne in mind that epitope peptides exhibit little immunogenicity and need to be used in combination with adjuvants, which increase immunogenicity by inducing strong innate immune responses that enable adaptive immunity [144–146]. Consequently, the discovery of new adjuvants is particularly relevant for epitope-based vaccines [146] and to that end, Nagpal et al. [147] developed a pioneered method that can predict the immunomodulatory activity of RNA sequences.

## Figures and Tables

**Table 1:** Selected T-cell epitope prediction tools available online for free public use.

Tool	URL	Method^1^	MHC class	A	S	T	P	Ref.
EpiDOCK	http://epidock.ddg-pharmfac.net	SB	II	—	—	—	—	[86]
MotifScan	https://www.hiv.lanl.gov/content/immunology/motif_scan/motif_scan	SM	I and II	—	X	—	—	—
Rankpep	http://imed.med.ucm.es/Tools/rankpep.html	MM	I and II	—	—	—	X	[26]
SYFPEITHI	http://www.syfpeithi.de/	MM	I and II	—	—	—	—	[23]
MAPPP	http://www.mpiib-berlin.mpg.de/MAPPP/	MM	I	—	X	—	X	[87]
PREDIVAC	http://predivac.biosci.uq.edu.au/	MM	II	—	—	—	—	[88]
PEPVAC	http://imed.med.ucm.es/PEPVAC/	MM	I	—	X	—	X	[63]
EPISOPT	http://bio.med.ucm.es/episopt.html	MM	I	—	X	—	—	[64]
Vaxign	http://www.violinet.org/vaxign/	MM	I and II	—	—	—	—	[89]
MHCPred	http://www.ddg-pharmfac.net/mhcpred/MHCPred/	QSAR	I and II	X	—	—	—	[34]
EpiTOP	http://www.pharmfac.net/EpiTOP	QSAR	II	X	—	—	—	[90]
BIMAS	https://www-bimas.cit.nih.gov/molbio/hla_bind/	QAM	I	X				[28]
TEPITOPE	http://datamining-iip.fudan.edu.cn/service/TEPITOPEpan/TEPITOPEpan.html	QAM	II	X	—	—	—	[32]
Propred	http://www.imtech.res.in/raghava/propred/	QAM	II	X	X	—	—	[91]
Propred-1	http://www.imtech.res.in/raghava/propred1/	QAM	I	X	X	—	X	[92]
EpiJen	http://www.ddg-pharmfac.net/epijen/EpiJen/EpiJen.htm	QAM	I	X	—	X	X	[82]
IEDB-MHCI	http://tools.immuneepitope.org/mhci/	Combined	I	X	—	—	—	[93]
IEDB-MHCII	http://tools.immuneepitope.org/mhcii/	Combined	II	X	—	—	—	[93]
IL4pred	http://webs.iiitd.edu.in/raghava/il4pred/index.php	SVM	II	—	—	—	—	[67]
MULTIPRED2	http://cvc.dfci.harvard.edu/multipred2/index.php	ANN	I and II	—	X	—	—	[62]
MHC2PRED	http://www.imtech.res.in/raghava/mhc2pred/index.html	SVM	II	—	—	—	—	[38]
NetMHC	http://www.cbs.dtu.dk/services/NetMHC/	ANN	I	X	—	—	—	[49]
NetMHCII	http://www.cbs.dtu.dk/services/NetMHCII/	ANN	II	X	—	—	—	[30]
NetMHCpan	http://www.cbs.dtu.dk/services/NetMHCpan/	ANN	I	X	—	—	—	[54]
NetMHCIIpan	http://www.cbs.dtu.dk/services/NetMHCIIpan/	ANN	II	X	—	—	—	[55]
nHLApred	http://www.imtech.res.in/raghava/nhlapred/	ANN	I	—	—	—	X	[94]
SVMHC	http://abi.inf.uni-tuebingen.de/Services/SVMHC/	SVM	I and II	—	—	—	—	[95]
SVRMHC	http://us.accurascience.com/SVRMHCdb/	SVM	I and II	X	—	—	—	[46]
NetCTL	http://www.cbs.dtu.dk/services/NetCTL/	ANN	I	X	X	X	X	[83]
WAPP	https://abi.inf.uni-tuebingen.de/Services/WAPP/index_html	SVM	I	—	—	X	X	[37]

^1^Method used for prediction of peptide-MHC binding. Keys for methods: SM: sequence motif; SB: structure-based; MM: motif matrix; QAM: quantitative affinity matrix; SVM: support vector machine; ANN: artificial neural network; QSAR: quantitative structure-activity relationship model; combined: tool uses different methods including ANN and QAM, selecting the more appropriate method for each distinct MHC molecule. The table also indicates whether the tools predict quantitative binding affinity (A), supertypes (S), TAP binding (T), and proteasomal cleavage (P); marked with an X in the affirmative case.

**Table 2:** Selected B-cell epitope prediction methods available for free online use.

Tool	Method	Server (URL)	Ref.
*Linear B cell epitope*			
PEOPLE	Propensity scale method	http://www.iedb.org/	[103]
BepiPred	ML (DT)	http://www.cbs.dtu.dk/services/BepiPred/	[110]
ABCpred	ML (ANN)	http://www.imtech.res.in/raghava/abcpred/	[111]
LBtope	ML (ANN)	http://www.imtech.res.in/raghava/lbtope/	[112]
BCPREDS	ML (SVM)	http://ailab.ist.psu.edu/bcpred/	[113]
SVMtrip	ML (SVM)	http://sysbio.unl.edu/SVMTriP/prediction.php	[114]
*Conformational B-cell epitope*			
CEP	Structure-based method (solvent accessibility)	http://bioinfo.ernet.in/cep.htm	[118]
DiscoTope	Structure-based method (surface accessibility and propensity amino acid score)	http://tools.iedb.org/discotope/	[119]
ElliPro	Structure-based method (geometrical properties)	http://tools.iedb.org/ellipro/	[121]
PEPITO	Structure-based method (physicochemical properties and geometrical structure)	http://pepito.proteomics.ics.uci.edu/	[122]
SEPPA	Structure-based method (physicochemical properties and geometrical structure)	http://lifecenter.sgst.cn/seppa/	[123]
EPITOPIA	Structure-based method (ML-naïve Bayes)	http://epitopia.tau.ac.il/	[125]
EPSVR	Structure-based method (ML-SVR)	http://sysbio.unl.edu/EPSVR/	[126]
EPIPRED	Structure-based method (ASEP, Docking)	http://opig.stats.ox.ac.uk/webapps/sabdab-sabpred/EpiPred.php	[129]
PEASE	Structure-based method (ASEP, ML)	http://www.ofranlab.org/PEASE	[130]
MIMOX	Mimotope	http://immunet.cn/mimox/helps.html	[131]
PEPITOPE	Mimotope	http://pepitope.tau.ac.il/	[132]
EpiSearch	Mimotope	http://curie.utmb.edu/episearch.html	[133]
MIMOPRO	Mimotope	http://informatics.nenu.edu.cn/MimoPro	[134]
CBTOPE	Sequence based (SVM)	http://www.imtech.res.in/raghava/cbtope/submit.php	[136]
